# The understanding of core pharmacological concepts among health care students in their final semester

**DOI:** 10.1186/s12909-015-0522-z

**Published:** 2015-12-29

**Authors:** Patrik Aronsson, Shirley Booth, Staffan Hägg, Karin Kjellgren, Ann Zetterqvist, Gunnar Tobin, Margareta Reis

**Affiliations:** 1 Department Pharmacology, Institution of Neuroscience and Physiology, Sahlgrenska Academy, University of Gothenburg, Gothenburg, Sweden; 2 Department Pedagogical, Curricular and Professional Studies, Faculty of Education, University of Gothenburg, Gothenburg, Sweden; 3 School of Education, University of the Witwatersrand, Johannesburg, South Africa; 4 Department of Medical and Health Sciences, Faculty of Health Sciences, Division of Drug Research/Clinical Pharmacology, Linköping University, 581 85 Linköping, Sweden

## Abstract

**Background:**

The overall aim of the study was to explore health care students´ understanding of core concepts in pharmacology.

**Method:**

An interview study was conducted among twelve students in their final semester of the medical program (*n* = 4), the nursing program (*n* = 4), and the specialist nursing program in primary health care (*n* = 4) from two Swedish universities. The participants were individually presented with two pharmacological clinically relevant written patient cases, which they were to analyze and propose a solution to. Participants were allowed to use the Swedish national drug formulary. Immediately thereafter the students were interviewed about their assessments. The interviews were audio-recorded and transcribed verbatim. A thematic analysis was used to identify units of meaning in each interview. The units were organized into three clusters: pharmacodynamics, pharmacokinetics, and drug interactions. Subsequent procedure consisted of scoring the quality of students´ understanding of core concepts. Non-parametric statistics were employed.

**Results:**

The study participants were in general able to define pharmacological concepts, but showed less ability to discuss the meaning of the concepts in depth and to implement these in a clinical context. The participants found it easier to grasp concepts related to pharmacodynamics than pharmacokinetics and drug interactions.

**Conclusion:**

These results indicate that education aiming to prepare future health care professionals for understanding of more complex pharmacological reasoning and decision-making needs to be more focused and effective.

## Background

Pharmacotherapy is a cornerstone in the treatment of many diseases and consequently a large proportion of the population is prescribed or self-medicates with pharmaceuticals [1]. Although a variable response to medications has long been acknowledged, drug therapy has generally employed a broad treatment approach to a heterogeneous group of patients instead of a unique treatment approach to an individual patient. Today the current opinion is, however, that pharmacotherapy should as far as possible be adjusted for each patient [2]. Thus, in the health care setting, more focus should be attributed to the monitoring and fine-tuning of drug treatment and to communicate pharmacological issues with individual patients.

During their pharmacological studies health care students (future physicians, nurses, and specialist nurses in primary health care, henceforth denoted “the students”) are exposed to generalized knowledge, which they must later put into context. The latter step has proven hard to master, thus hampering the students’ abilities to identify and handle complex medication treatments and, in the end, make qualified judgments and communicate these with patients [3]. Students often struggle when it comes to understanding the subject of pharmacology. It requires, among other things, that chemical, physiological and mathematical thinking are integrated. Because different groups of health care personnel have different skills and roles, overall understanding and communicative skills in pharmacokinetics/pharmacodynamics are essential to avoid the risk of incorrect use of medicines [4].

Interactions between simultaneously used drugs are common causes of undesirable drug-induced events in patients [5] such as adverse drug effects, insufficient efficacy or even drug intoxications. These effects might be explained by the prescriber’s limited knowledge of integrated pharmacology resulting in an inability to customize a drug’s pharmacokinetic or pharmacodynamics properties to a patient’s specific conditions [6, 7]. Apart from inter-professional communication and understanding which are crucial, if the healthcare professional does not have adequate pharmacokinetic and pharmacodynamic insights, trustworthy information may not be given to the patient. On the other hand if the physician/nurse is fully aware of the pharmacology of the prescribed drugs and the patient still does not understand, the explanation might be drawn from inadequacies in the communication.

Finally, even though the different disciplines have separate roles within the health care setting, inter-professional communication is required to maintain the quality of care. The outcomes required to obtain the “Degree of Bachelor of Science in Nursing” (Nurse), “Postgraduate Diploma in Specialist Nursing: Primary Health Care” and “Degree of Master of Science in Medicine” (physician) are specified in Swedish law [8]. Here, it is clearly stated that all three professions are required, to master relevant aspects of drugs and drug treatments. For instance, a graduated nurse shall “demonstrate the ability to manage pharmaceuticals appropriately and also to inform patients of the effects and side-effects of pharmaceuticals” as well as to “demonstrate the ability to apply his or her knowledge to deal with different situations, phenomena and issues on the basis of the needs of individuals and groups”. Furthermore, graduated specialist nurses in primary health care, who have (limited) prescription rights, shall “demonstrate the ability to observe and assess complex care, habilitation and rehabilitation needs in patients”. The requirements for medical graduates are that they should “demonstrate the ability to initiate and undertake health promotion and preventive measures in the health care services for both individuals and groups of patients” and “demonstrate the ability to integrate and apply knowledge critically and systematically and also to analyze and assess complex phenomena, issues and situations” (translations from The Swedish Council for Higher Education; https://www.uhr.se/en/start/laws-and-regulations/Laws-and-regulations/The-Higher-Education-Ordinance/Annex-2/; accessed 2015-02-18). Thus, while some differences between the three cohorts studied regarding the depth of expertise obtained during their training may be present, the competences needed to deal with the cases presented could be expected from all three student groups.

**Fig. 1:**
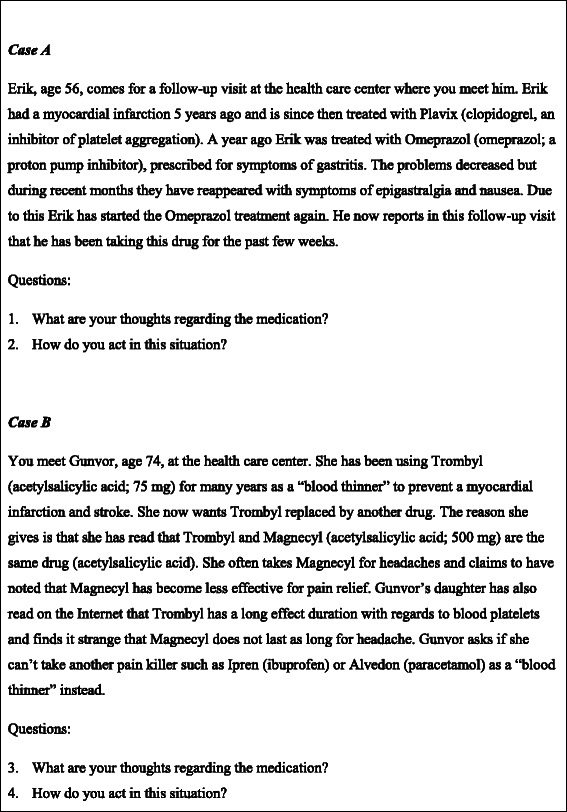
The two patient cases that were presented to the interviewees for discussion. *Case A* The patient is prescribed omeprazole (a proton pump inhibitor) in combination with the prodrug clopidogrel, an inhibitor of platelet aggregation (P2Y_12_ purinoceptor antagonist). Since clopidogrel is activated by an enzyme (CYP2C19) that is inhibited by omeprazole possible effects of an interaction may appear, which should be considered by the interviewed. *Case B* The patient is prescribed acetylsalicylic acid (at a low dose) because of cardiovascular events. The patient wants it to be replaced by another non-steroid, anti-inflammatory drug (NSAID) or paracetamol (acetaminophen). The discussion of this case requires the understanding of the mechanism of action of NSAIDs (reversible and irreversible cyclooxygenase binding) and its relevance for the dosage

The overall aim of the current study was to explore health care students’ understanding of core concepts in pharmacology. Specifically the following questions were asked: 1. How do students undertaking nursing and medical educations understand core concepts of pharmacokinetics, pharmacodynamics and drug interactions? 2. How do the students adapt their pharmacological knowledge and apply the understanding of the core concepts of pharmacokinetics, pharmacodynamics and drug interactions when presented with two clinically relevant patient cases? (Fig. 1)

.

## Methods

### Study subjects

Twelve students in their final semester from the medical program, henceforth abbreviated DOC, nursing program (RN), and the specialist nursing program in primary health care (PHCN), were recruited to the study; four students from each category from two medical schools in Southern Sweden (Table 1). There was no intention to compare the performance of students from the different universities. The spread of backgrounds was intended to give a qualitative variation of the answers given. Students were asked to voluntarily sign up for participation in the study. All interviewees gave their informed consent prior to their inclusion in the study. The current study does not involve any handling of sensitive personal data or clinical procedures and therefore no ethical review is required by law in Sweden according to the Act (2003:460) concerning the Ethical Review. The project therefore complies fully with current applicable Swedish legal rules and ethical guidelines including the Helsinki declaration.

**Table 1:** Describing participating students; primary healthcare nurse students (PHCN), medical students (DOC) and nurse students (RN)

Future profession	Student number
PHCN	1, 2, 3, 4
DOC	5, 6, 7, 12
RN	10, 11, 13, 14

### Case introduction

The students were individually presented with two written cases describing patients treated with a combination of drugs. The cases were designed to imitate common health care provider-patient interactions. This allows the students to analyze the problems and to apply their pharmacological knowledge, acquired from their respective educations, in combination with their information collecting skills. Thereby, the depth of the understanding of the posed problems (Fig. 1) would be revealed. The first case mainly addressed drug problems related to pharmacodynamic issues, while the second was directed towards pharmacokinetic-related problems. The present paper focuses on the students’ understanding and ability to articulate the pharmacological concepts pharmacodynamics, pharmacokinetics and drug interactions. In a paper we recently published, we investigated and problematized the pharmacological communication within the same setting [9]. The study participants did not have access to the internet and the only tool provided was a hardcopy of FASS, the Swedish national drug formulary which provides healthcare professionals with detailed information about approved pharmaceuticals. During 30 min (15 min per case) the student was expected to identify the pharmacological problems and find possible solutions. Immediately thereafter, the students discussed the cases for approximately 30 min individually with an interviewer (MR). The interviews were semi-structured. All interviewees were presented with the same patient cases and asked the same opening questions (Fig. 1) to clarify the meaning of the answers. The intention was to explore the interviewees´ understanding of the cases given. In the conversation following the opening questions the student´s understanding of the concepts of pharmacokinetics, pharmacodynamics and drug interactions were elicited. All interviews were audio-recorded and subsequently transcribed into text. Drugs were referred to primarily by brand names, rather than generic names, because of the structure of the FASS drug formulary.

### Interview analysis

**Fig. 2:**
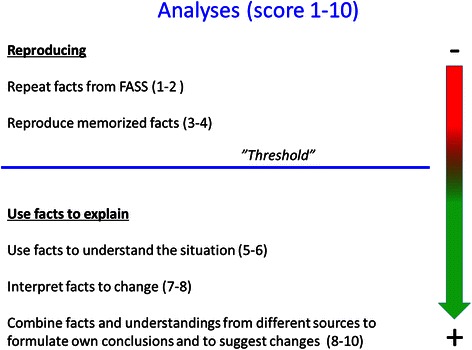
In the analysis of unites of meaning the quality of each unit was awarded a score from 1–10. The figure displays the competences expected on each level

A thematic analysis was used [10] for identifying and analyzing patterns or themes in the interviews. The method allows for working with both a deductive and inductive approach to the data. The inductive approach (i.e. without trying to fit into a predefined coding frame) and the deductive approach (i.e. driven by our analytic interest) were used iteratively. The analysis consisted of identifying units of meaning which are coherent and distinct meanings embedded within the interviews [11]. A unit of meaning may consist of one word or several sentences. The units were organized in three thematic clusters: pharmacodynamics, pharmacokinetics, and drug interactions. In a subsequent analysis the quality of the extracted units of meaning were rated with scores 1–10 (Fig. 2). Two of the authors (GT and MR), independently, assessed each unit of meaning in all interviews. The analysis was iterative, and the findings were reviewed and discussed in order to reach agreement in understanding of the data.

### Statistics

Non-parametric statistics was used and Kruskal-Wallis analysis was employed when three groups were compared.

## Results

**Fig. 3:**
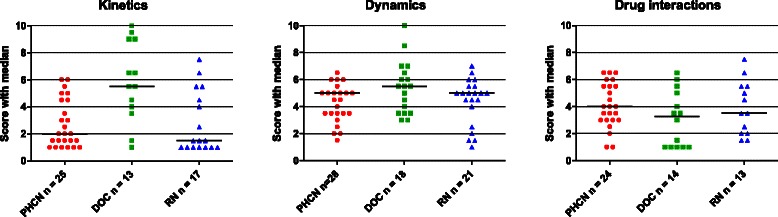
Graphic overview of the results of the quality of units of meaning (score 1–10 and median). Results related to the core concepts of pharmacology; pharmacokinetics, pharmacodynamics and drug interactions, subdivided by groups of future health care professionals, i.e. primary healthcare nurse students (PHCN; ●), medical students (DOC; ■) and nurse students (RN; ▲). *n* = Number of units of meanings expressed by each category of future professionals. All participants contributed in each case

All 12 students completed the study. When analyzing the units of meaning a great variation in students´ answers was observed with regard to the interpretation of the task, the elaboration and the quality of the reflections. Results of the quality assessment of units of meaning are graphically presented in Fig. 3. Each student´s result, including number and quality of statements (scores 1–10), is displayed in Table 2. Below quotes are organized to exemplify, in the first part, variation in quality of reflections and then to illustrate the understanding of the core concepts: pharmacodynamics; pharmacokinetics; and drug interactions.

**Table 2:** Number of units of meaning (UM) expressed per student on the subjects kinetics, dynamics, and drug interaction. In addition, the median score value per student on respective subject is presented

*PHCN*	UM	Score	DOC	UM	Score	RN	UM	Score
*student*	(n)	median	student	(n)	median	student	(n)	median
Kinetics								
* 1*	4	5.25	*7*	4	4.75	*13*	3	1
* 2*	6	1	*12*	3	9	*14*	4	1.25
* 3*	3	3	*5*	3	6.5	*10*	4	3.5
* 4*	12	2	*6*	3	1.5	*11*	6	4.25
Dynamics								
* 1*	5	4.5	*6*	8	3.5	*13*	4	6
* 2*	4	2.75	*7*	5	7	*14*	4	4.5
* 3*	6	5	*12*	3	6.5	*10*	5	5
* 4*	13	5	*5*	2	4.5	*11*	8	5
Interaction								
* 1*	8	5.75	*7*	2	6	*13*	3	1.5
* 2*	4	4	*12*	2	4.25	*14*	6	4
* 3*	5	3.5	*5*	4	1.25	*10*	1	2.5
* 4*	7	3.5	*6*	6	2	*11*	3	5

Although most students were able to define terms like half-life, clearance, distribution volume etc. within the cases, a number of units of meaning revealed an insufficient understanding even regarding such important terminology. The following two quotes illustrate their uncertainty:

*“Half-life? Well, that’s tricky. I don’t know if I remember exactly what sort of stuff that is, ‘cause I failed that topic on the exam last time…..”* (Student No. 2)

and

*“I: What do you include into the concept metabolite?*

*R: It is probably the way it acts…”* (Student No. 11)

Such statements are in contrast to the discussion by other students where they merge and interpret pharmacodynamic and pharmacokinetic information in order to explain the situation of the patient.

*“Among other things that CYP2C19 was the common denominator… both go via the CYP450 system but… it is metabolising if you consider these CYPs, and that in FASS they specifically dissuades from combining omeprazole and Plavix (clopidogrel) treatments. It has been observed that as much as 45 % of the active aggregation is inhibited, well, Plavix is inhibited.”* (Student No. 12)

The overall evaluation of the units of meaning shows that the students used the pharmacodynamic information at a more advanced level than statements regarding pharmacokinetics and drug interactions. In the latter cases, the participants more often made just a simple definition of the words. Generally, the students tended to stress the pharmacodynamic topics of the patient cases over the pharmacokinetic or interaction issues. When comparing the median scores reached per pharmacokinetic (PK), pharmacodynamic (PD), and interaction (I) units of meaning within each professional category, pharmacodynamic units of meaning reached the highest scores for all students; significantly for PHCN (median values: PK 2; PD 5; I 4; *P* = 0.002) and RN students (PK 1.5; PD 5; I 3.5; *P* = 0.04). For DOC students, however, pharmacodynamic and pharmacokinetic units scored equal whereas units regarding interactions scored lower (PK 5.5; PD 5.5; I 3.2; *P* = 0.01). Further, when median scores for pharmacokinetic, pharmacodynamic, and interaction units of meaning were compared between the professional categories, DOC scored significantly higher for pharmacokinetics than did PHCN or RN (PHCN 2; DOC 5.5; RN 1.5; *P* = 0.005). No differences were seen between the students for pharmacodynamics or drug interactions. Finally, in a comparison of median scores reached per core pharmacological concept, pharmacodynamics reached the highest scores, regardless of profession (PK 2.5; PD 5; I 3.5; *P* = 0.002). For example, in spite of omeprazole and clopidogrel interacting at a metabolic level, this was usually not mentioned even though the efficacy of clopidogrel was discussed.

*“Yes… that they interact in some way… I think it is said that the effect of omeprazole was less good.”* (Student No. 9)

## Pharmacodynamics

The participants focused, almost without exception, their discussions of the cases on the coupling between the drugs and their pharmacodynamic effects on disease. Most participants realized the importance of dosage for the desired effect, as well as for the risks of adverse effects. Often the effect of the drugs on the disease was discussed, without any reflection on the mechanisms of action. For instance, only a few participants discussed the mechanisms behind the differences in action of the aspirin-exerted cyclooxygenase (COX)-inhibition in pain compared to blood clotting. Some participants brought up the fact that not only the dose, but also the duration of the effect on the platelets is of importance and one student suggested incorrect different durations of action between tablets containing different dosages of acetylsalicylic acid.

*“… I read that Trombyl (acetylsalicylic acid, 75 mg) and Magnecyl (acetylsalicylic acid, 500 mg) both consist of acetylsalicylic acid, which both inhibits the platelet aggregation, but that Trombyl more strongly inhibits the platelet aggregation and has a long-lasting duration for seven to ten days….”* (Student No. 10)

Most did, however, not make this consideration but regarded the drug effects to be purely dose-correlated without any confounding factors.

*“…and that Magnecyl (acetylsalicylic acid), I think is 500 mg, while Trombyl (acetylsalicylic acid) is 75 mg, which means it is huge dose of blood-thinning in Magnecyl and I think this is generally overlooked by patients. I hadn’t given this a thought before reading it. So, well, and all these unwanted effects, which appear mostly from acetylsalicylic acid but also from other NSAIDs as well.”* (Student No. 11)

and

*“Well… she mentions them both here, that it is the same substance, acetylsalicylic acid for instance, and both are blood-thinners but act in two different ways. Or Trombyl (acetylsalicylic acid 75 mg) is blood-thinning, which she takes in order to prevent stroke and heart infarction and Magnecyl (acetylsalicylic acid 500 mg) is the actual painkiller even though it also has a blood-thinning effect.”* (Student No. 2)

and

*”… and inform that Trombyl (acetylsalicylic acid 75 mg) and Magnecyl (acetylsalicylic acid 500 mg) are actually the same thing, but is used for different reasons and that this is a matter of dose, then I would also try to explain that they have somewhat different effects in the body, they inhibit inflammation and pain but affect the platelets as well and because the blood-cells live longer, or live for a long time and that prostaglandins are produced constantly, they can have a long-lasting effect on some things but a shorter effect on other things…”* (Student No. 7)

Few of the participants realized that Plavix (clopidogrel) needed to be activated in order to exert its pharmacological effect.

*“But he needs some sort of medicine for this and in FASS they say that there exists an interaction between Plavix (clopidogrel) and omeprazole which reduces the active metabolites”* (Student No. 7)

### Pharmacokinetics

In contrast to the pharmacodynamic information in the presented cases, the participants generally seemed to find it hard to realize the significance of the pharmacokinetics of the drugs used by the fictive patients. This is reflected by statements indicating that using only pharmacodynamic aspects of the drugs is sufficient to explain the whole situation, which generally is not the case.

*“No. I chose actively not to do it [read about it pharmacokinetics] since the whole story seems blurry and I think it is better to straighten out what it´s all about before one digs in into the details.”* (Student No. 6)

The clinical relevance of pharmacokinetic data seems still to be obscure for many participants even though they may understand the basic meaning of the concepts.

*“The half-life is important in order to get a good dosage regimen, to get a constant flow and in some way they are merged together so that one does not reflect as intensively about it, it just goes with the flow when you read the text… But I do not look into it specifically or reflect about it.”* (Student No. 12)

Only very few made interpretations of the pharmacokinetic data by putting the given situation into new perspectives. As written previously, the DOC students reached higher scores for pharmacokinetic statements than did the other students.

*“Well, the pharmacokinetics depend on what uptake, metabolism and excretion you have, so there I thought that he could actually suffer from some newly received disease or something like that, which could in fact result in a higher concentration of clopidogrel.”* (Student No. 5)

### Drug interactions

When considering the polypharmacy issues of the cases, problems with drug interactions were often considered according to the pharmacodynamic effects.

*“Since they act on the same systems, both consist of acetylsalicylic acid, and both will inhibit the platelets, so they will potentiate each other’s effect, there will be a double effect on the platelets.”* (Student No. 6)

However, some participants considered pharmacokinetic aspects regarding drug interactions.

*“It is quite unclear why omeprazole inhibited Plavix (clopidogrel) but it was… most things indicated that it was via CYPC19.”* (Student No. 7)

The analysis revealed that most statements made were of a reproducing nature and only a few displayed a deeper understanding of core pharmacology concepts. This may be depicted in Fig. 3 indicating that the median ratings rarely were above the threshold. In general, the students seldom made any further extrapolation of the information for more in-depth discussions.

## Discussion

Generally, the participants in this study focused on defining the concepts (pharmacodynamics, pharmacokinetics and interactions) and only to a much lesser extent used them in further discussions. In the instances when a more advanced discussion was initiated, the emphasis was on pharmacodynamic aspects, rather than also taking pharmacokinetic properties into consideration. Furthermore, it was largely the clinical effects of the drugs that were looked into, whereas mechanisms of action etc. were rarely scrutinized. Also, the students had some capability to extrapolate the meaning of the concepts in pharmacodynamics and pharmacokinetic aspects when discussing only one drug at a time. However, only few, and then often erroneous, statements and interpretations were made relating to drug interactions. Overall, the students seem to have a low ability to apply basic pharmacological concepts practically in complex conditions.

Students studying pharmacology often tend to focus on understanding the mechanism of action of the drug, whilst the pharmacokinetic course content attracts less interest [12], which is in line with our results. Pharmacokinetics is often overlooked, in spite of its necessity for fully grasping the application of pharmacology in the clinical situation. The similarity in ability to discuss pharmacodynamic and drug interaction issues is not entirely unexpected. Students consider pharmacodynamic reasoning to be more intuitive and easier to grasp than pharmacokinetic reasoning [13]. Drug interactions may, in this case, be seen as a “mix” of pharmacokinetic and pharmacodynamic factors interacting. This interplay is necessary for the student to grasp in order to realize how to achieve optimal dosing [3, 4].

When interpreting the results of the current study, one must take into consideration that the number of participants was quite small and that the selection of the students was not randomized. Rather, students were approached and only those showing interest in participating in the study were included. One may argue that this would, perhaps, provide the study with “stronger students”, eager to discuss these matters. However, this hypothesis generating study seemed to have functioned well, since the presented cases triggered all participants to respond irrespective of their education. One reason is probably that they were presented with two clinically realistic cases of the kind they would meet in their future everyday work including layman language like “blood-thinner”.

And in spite of the rather few participants, each one was able to relate, in one way or another, to the cases, which resulted in lively discussions rendering a high total number of statements. In the analysis method that we applied, all data were considered as one set of transcripts [14] and by that a large number of statements (units of meaning) could be analyzed. Furthermore, it was rather obvious that even though the participants were in the final semester of their education they considered the interview to be something of an examination, in which they were eager to provide the interviewer with correct answers. This may have hampered their motivation to further expand and deepen the discussion into unknown areas. Despite this, if one may consider the current results to reflect a true situation, we conclude that the students have an almost acceptable level of knowledge about the core concepts, but that they obviously fail in their capability of integrating them in a pharmacological interpretation.

The results of the current study indicate a gap in the education between the introduction of the core concepts of pharmacology and the application of these concepts in a clinical context, even though the small sample size employed in the present investigation necessitates caution when attempting to extrapolate the results to all health care students. Since the curricula in all programs represented in the study include both the parts; introduction of concepts and patient cases, the translation of the concepts into a clinical context is potentially a key issue [13, 15]. Other studies also indicate that the latter step is important for a more functional understanding of pharmacology concepts [16]. Irrespective of pedagogic approach, more multifaceted pharmacological reasoning and decision-making ought to be expanded and optimized, with a progressive complexity within respective curricula. Not only must the extent of the course time allocated for the basic medical subjects be, if not increased, at least maintained at the current level. If these concepts are indeed considered important to master in a modern complex and individualized health care setting, then efforts must be made to encourage students to discuss and analyze pharmacological issues beyond a superficial level. Pharmacological knowledge that takes account of specific issues regarding pharmacokinetics and pharmacodynamics will promote long-term health, safety, ethics and health economics.

## Conclusions

In general, the final semester students in this study were able to define pharmacological concepts, but showed less ability to discuss the meaning of the concepts in depth and to interpret the consequences of the given information in a clinical context. The participants seemed to experience pharmacodynamic data to be easier to grasp than pharmacokinetics. These results indicate that education aiming to prepare future health care professionals for more complex pharmacological reasoning and decision-making should probably be more focused and effective.

## Abbreviations

PK: Pharmacokinetics
PD: Pharmacodynamics
I: Drug interaction
RN: Students from nursing program
PHCN: Students from specialist nursing program in primary health care
DOC: Students from the medical program
